# Dose-Dependent Mutation Rates Determine Optimum Erlotinib Dosing Strategies for EGFR Mutant Non-Small Cell Lung Cancer Patients

**DOI:** 10.1371/journal.pone.0141665

**Published:** 2015-11-04

**Authors:** Lin L. Liu, Fei Li, William Pao, Franziska Michor

**Affiliations:** 1 Department of Biostatistics, Harvard T. H. Chan School of Public Health, Boston, MA 02115, United States of America; 2 Department of Biostatistics and Computational Biology, Dana-Farber Cancer Institute, Boston, MA 02215, United States of America; 3 Vanderbilt-Ingram Cancer Center, Nashville, TN 27232, United States of America; University of Catania, ITALY

## Abstract

**Background:**

The advent of targeted therapy for cancer treatment has brought about a paradigm shift in the clinical management of human malignancies. Agents such as erlotinib used for EGFR-mutant non-small cell lung cancer or imatinib for chronic myeloid leukemia, for instance, lead to rapid tumor responses. Unfortunately, however, resistance often emerges and renders these agents ineffective after a variable amount of time. The FDA-approved dosing schedules for these drugs were not designed to optimally prevent the emergence of resistance. To this end, we have previously utilized evolutionary mathematical modeling of treatment responses to elucidate the dosing schedules best able to prevent or delay the onset of resistance. Here we expand on our approaches by taking into account dose-dependent mutation rates at which resistant cells emerge. The relationship between the serum drug concentration and the rate at which resistance mutations arise can lead to non-intuitive results about the best dose administration strategies to prevent or delay the emergence of resistance.

**Methods:**

We used mathematical modeling, available clinical trial data, and different considerations of the relationship between mutation rate and drug concentration to predict the effectiveness of different dosing strategies.

**Results:**

We designed several distinct measures to interrogate the effects of different treatment dosing strategies and found that a low-dose continuous strategy coupled with high-dose pulses leads to the maximal delay until clinically observable resistance. Furthermore, the response to treatment is robust against different assumptions of the mutation rate as a function of drug concentration.

**Conclusions:**

For new and existing targeted drugs, our methodology can be employed to compare the effectiveness of different dose administration schedules and investigate the influence of changing mutation rates on outcomes.

## Introduction

Recent advances have improved our understanding of the molecular alterations that drive particular cancer types and have thus enabled the development of targeted agents that specifically inhibit these lesions [1]. Examples of targeted therapies include small molecule inhibitors of the epidermal growth factor receptor (EGFR) pathway in lung cancer (e.g. erlotinib (Tarceva)) and inhibitors of the BCR-ABL tyrosine kinase in chronic myeloid leukemia (e.g. imatinib (Gleevec), dasatinib (Sprycel), and nilotinib (Tasigna)). These small molecule inhibitors are taken up into cancer cells where they interfere with abnormal signaling. Targeted therapy differs from traditional cytotoxic chemotherapy in that it not only leads to more specific effects with reduced toxicity, but also promises a future of personally tailored anti-cancer treatment [2].

The development of targeted anti-cancer therapies requires the design of optimal treatment strategies so that responses are maximized while toxicity remains tolerable [3]. Because of the combinatorial complexity of this problem, systematic and mathematical approaches have been employed in the past to identify best treatment modalities. In a seminal paper in 1977, Norton and Simon proposed a model of kinetic (i.e., non-genetic) resistance to cell-cycle specific therapy in which tumor cells followed a Gompertzian growth law [4]. This work led the authors to propose a dose-intensification strategy, supported with historical data [5] and later on Implemented as a prospective clinical trial [6]. Their model and its predictions have become known as the Norton-Simon hypothesis and inspired many subsequent studies of kinetic resistance [7–14]. In parallel, several investigations addressed the emergence of genetic resistance, i.e. resistance driven by genetic alterations in cancer cells. Coldman and co-authors were the first to introduce stochastic models of resistance against chemotherapy to guide treatment schedules [15], which led to many subsequent studies by these authors [15–17] and others [18–28]. Several other papers addressed the question about optimum dosing of targeted therapy by including the effect of quiescence on the kinetics of response to treatment [29, 30]. We recently developed a stochastic framework to optimize dosing strategies of targeted drugs [31, 32]; when applied to EGFR-mutant non-small cell lung cancer, this model allowed us to identify a treatment schedule predicted to maximally delay the onset of T790M-driven resistance [33], which is the most common mechanism of disease progression. This schedule is currently being validated in a clinical trial at Memorial Sloan-Kettering Cancer Center (NCT01967095), in which patients receive high dose erlotinib on day 1 and day 2 with daily low dose erlotinib on days 3 through 7. Dose levels for the high dose pulse are escalated starting from 600mg oral daily on day 1 and day 2 until the maximally tolerated dose is reached. First results are expected in 2016.

In this paper, we aim to extend these investigations by taking into account the ability of a targeted drug to alter the kinetics of mutation acquisition in cancer cells: the rate at which resistant cells emerge might now depend on the dose of drug administered to the patient. A possible mechanism driving this phenomenon might be the fact that DNA damage can result from the intermediates of oxygen reduction in a cell. Reactive oxygen species (ROS) are short-lived oxygen intermediates and are generated primarily by the mitochondrial respiratory chain in inflammatory cells. By interacting with free nucleotides, ROS promotes DNA damage [34], which can subsequently result in a higher frequency of mutations in the cell. Koptyra et al. [35], for instance, showed that by inhibiting ROS in leukemia cells, the mutagenesis rate was decreased, which in turn resulted in a decreased frequency of resistance against imatinib. Recent studies on the effects of traditional cytotoxic drugs have also revealed a connection between ROS and induced apoptosis in cancer cells [36]. Furthermore, traditional cytotoxic drugs were found to be associated with the generation of ROS [37–39]. The administration of anti-cancer drugs, both traditional chemotherapeutic agents and targeted drugs, may thus modulate the rate at which mutations arise in cancer cells, thereby influencing the dynamics of resistance. Here we derive a quantitative framework of dynamic mutation rates during the treatment of cancer and use this framework to identify best treatment modalities for the clinical management of human malignancies.

## Methods

**Fig 1:**
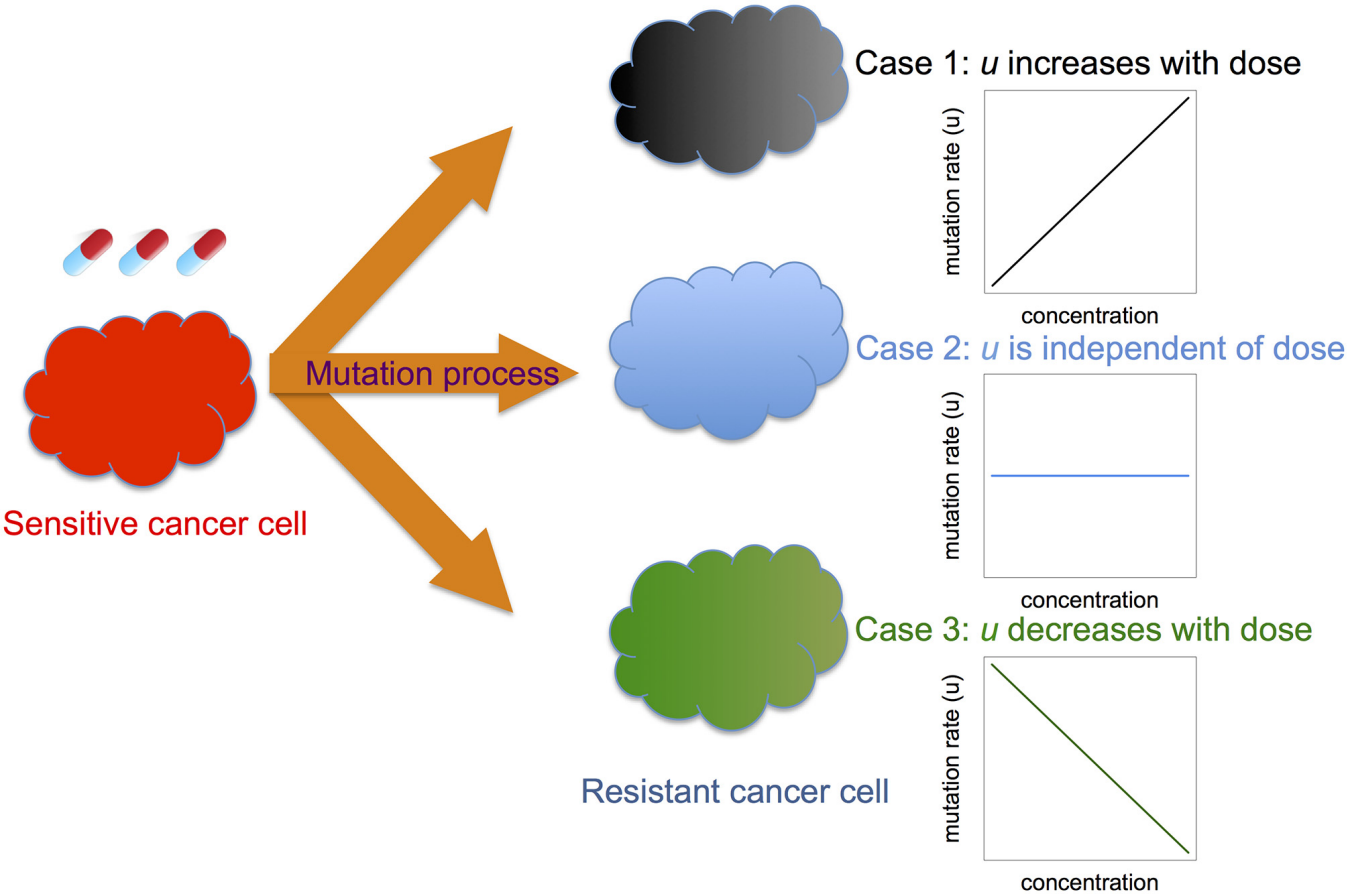
Schematic overview of the model. We developed a mathematical model to investigate the effects of changing mutation rates during treatment on the evolution of resistance. When treated with targeted or traditional cytotoxic chemotherapy, a sensitive cancer cell (left) might give rise to a resistant cell at a rate that increases with the drug dose administered (case 1), is independent of the dose (case 2), or decreases with the dose (case 3). For simplicity, we show linear relationships between drug dose and mutation rate; however, more complex relationships can easily be considered using our general framework.

Consider a population of tumor cells proliferating within a tissue. We model the evolutionary dynamics of the tumor population as a multi-type non-homogeneous continuous-time birth-death stochastic process. The population consists of two types of cells: sensitive and resistant cancer cells. These cells might describe the small subset of cells capable of propagating the entire tumor cell population (i.e., “cancer stem cells”) or alternatively the entire tumor mass (i.e.“tumor bulk”). At any given time point *t*, the number of sensitive cancer cells is denoted as *X*_*s*_(*t*), whereas the number of resistant cancer cells is represented by *X*_*r*_(*t*). Sensitive cancer cells proliferate at rate *λ*_*s*_(*t*) and die at rate *μ*_*s*_(*t*). During each sensitive cell division, a mutation that confers resistance occurs at probability *u*(*t*); this quantity might depend on time as well. Resistant cancer cells proliferate and die at rates *λ*_*r*_(*t*) and *μ*_*r*_(*t*). Note that we use *λ*_*s*_(*t*), *λ*_*r*_(*t*), *μ*_*s*_(*t*), *μ*_*r*_(*t*), and *u*(*t*) as *per capita* rates or probabilities per time unit. The possibility of back-mutation from resistant to sensitive cells is excluded. Treatment alters growth and/or death rates of sensitive and potentially also resistant cells, thereby modulating these rates over time depending on the treatment schedule used (Fig 1).

We define the birth-death process as ***X***(*t*) ≡ (*X*_*s*_(*t*),*X*_*r*_(*t*)). Let us first consider situations in which the initial cancer population merely consists of *M* sensitive cells, i.e. ***X***(*t*) = (*M*, 0). This assumption will be relaxed in later sections to better describe biological situations [40, 41]. This stochastic process is defined by the following infinitesimal transition probabilities:

$$\begin{array}{c} \begin{array}{cc} & P(X(t+\Delta t)=(n+j,m+k)|X(t)=(n,m))=\\ & \left\{\begin{array}{cc} \lambda_{s}\left(t\right)n\Delta t\left(1-u\left(t\right)\right)+o\left(\Delta t\right) & \;\text{if}\;j=1,k=0,\\ \mu_{s}\left(t\right)n\Delta t+o\left(\Delta t\right) & \;\text{if}\;j=-1,k=0,\\ \lambda_{r}\left(t\right)m\Delta t+\lambda_{s}\left(t\right)n\Delta tu\left(t\right)+o\left(\Delta t\right) & \;\text{if}\;j=0,k=1,\\ \mu_{r}\left(t\right)m\Delta t+o\left(\Delta t\right) & \;\text{if}\;j=0,k=-1,\\ 1-\left(\lambda_{s}\left(t\right)+\mu_{s}\left(t\right)\right)n\Delta t-\left(\lambda_{r}\left(t\right)+\mu_{r}\left(t\right)\right)m\Delta t+o\left(\Delta t\right) & \;\text{if}\;j=0,k=0,\\ o(\Delta t) & \;\text{else.} \end{array}\right. \end{array} \end{array}$$ (1)

Let us now derive some quantities of interest including the expected number of resistant cells, the variance of the number of resistant cells, and the probability of resistance. In all cases, we generalize our calculations to cases with a pre-existing resistant clone before treatment. The cases without any pre-existing resistant clones can be derived by setting *X*_*r*_(0) = 0 for all following equations.

To perform exact computer simulations of this process, we employed adaptive thinning to overcome the difficulties resulting from time-dependent birth, death and mutation rates [42]. In this algorithm, exponential waiting times between events are generated by first calculating a constant rate which majorizes the true instantaneous rate at any time *t*. Then, for each event, an exponential waiting time is generated with this majorizing rate. This event is accepted if a uniform random variable generated on [0,1] falls below the ratio of the true rate to the majorizing rate; otherwise, the event is rejected. The simulations are used to validate the approximations developed below.

### The expected number of resistant cells

Since the mutation rate is always much less than 1, *u* < < 1, we can approximate the birth rate of sensitive cells, *λ*_*s*_(1−*u*), with *λ*_*s*_ in the derivations of the important characteristics of our model. We will later verify the validity of this approximation through the consistency of our theoretical derivations with the exact stochastic computer simulations; see the Results section. Let us first calculate the rate of transition from sensitive to resistant cells, given by

$$\begin{array}{c} r\left(t\right)=X_{s}\left(0\right)\cdot exp\left\{\int_{0}^{t}\left[\lambda_{s}\left(\tau \right)-\mu_{s}\left(\tau \right)\right]d\tau \right\}\cdot \lambda_{s}\left(t\right)u\left(t\right). \end{array}$$ (2)

Then the expected number of resistant cells arising from the sensitive cell population is given by

$$\begin{array}{c} E\left[X_{sr}\left(t\right)\right]=\int_{0}^{t}r\left(\tau \right)\cdot exp\left\{\int_{0}^{t-\tau }\left[\lambda_{r}\left(\tau +\eta \right)-\mu_{r}\left(\tau +\eta \right)\right]d\eta \right\}d\tau . \end{array}$$ (3)

Hence the expected number of resistant cells at time *t* is given by

$$\begin{array}{l} E\left[X_{r}\left(t\right)\right]\;=E\left[X_{rr}\left(t\right)\right]+E\left[X_{sr}\left(t\right)\right]\\ \;=X_{r}\left(0\right)exp\left\{{{\int^{\text{​}}}^{\;}}_{0}^{t}\left[\lambda_{r}\left(\tau \right)-\mu_{r}\left(\tau \right)\right]d\tau \right\}+{{\int^{\text{​}}}^{\;}}_{0}^{t}r\left(\tau \right)exp\left\{{{\int^{\text{​}}}^{\;}}_{0}^{t-\tau }\left[\lambda_{r}\left(\tau +\eta \right)-\mu_{r}\left(\tau +\eta \right)\right]d\eta \right\}d\tau . \end{array}$$ (4)

where *X*_*rr*_(*t*) is the number of resistant cells generated from resistant cells at time *t*, and *X*_*sr*_(*t*) is the number of resistant cells generated from sensitive cells at time *t* respectively.

### The probability of resistance

Another quantity of interest is the probability that there is at least one resistant cell present at time *t*. For an infinitesimal time interval [*t*_*i*_,*t*_*i*_ + Δ*t*], the rate of emergence of resistant cells is given by *r*(*t*_*i*_)Δ*t*. As shown previously [32], under the assumption of no back mutation, the probability of resistance arising from the sensitive cell population is given by

$$\begin{array}{cc} P_{r}\left(T\right)\; & =1-P_{0}\left(T\right)\\ & =1-P_{0}^{s}\left(T\right)P_{0}^{r}\left(T\right)\\ & =1-exp\left\{\overset{T}{\underset{0}{\int }}r\left(t\right)P_{ext}\left(t,T\right)-r\left(t\right)dt\right\}{\left[\frac{\int_{0}^{T}\mu_{r}\left(t\right)exp\left\{\int_{0}^{t}\left[\mu_{r}\left(\tau \right)-\lambda_{r}\left(\tau \right)\right]d\tau \right\}dt}{1+\int_{0}^{T}\mu_{r}\left(t\right)exp\left\{\int_{0}^{t}\left[\mu_{r}\left(\tau \right)-\lambda_{r}\left(\tau \right)\right]d\tau \right\}dt}\right]}^{X_{r}\left(0\right)}, \end{array}$$ (5)

where *P*_0_(*T*) denotes the probability of having no resistant cells at time *T*, $P_{0}^{s}\left(T\right)$ the probability of having no resistant cells at time *T* that is generated from the sensitive clone, $P_{0}^{r}\left(T\right)$ the probability that the clone arising from the initial resistant cell has become extinct by time *T*, and

$$\begin{array}{c} \begin{array}{c} P_{ext}\left(t,T\right)=\frac{\int_{0}^{T-t}\mu_{r}\left(\tau +t\right)exp\left\{\int_{0}^{\tau }\left[\mu_{r}\left(\eta +t\right)-\lambda_{r}\left(\eta +t\right)\right]d\eta \right\}d\tau }{1+\int_{0}^{T-t}\mu_{r}\left(\tau +t\right)exp\left\{\int_{0}^{\tau }\left[\mu_{r}\left(\eta +t\right)-\lambda_{r}\left(\eta +t\right)\right]d\eta \right\}d\tau }. \end{array} \end{array}$$ (6)

### The variance of the number of resistant cells

The variance of *X*_*r*_(*T*) is also of interest because it provides a sense of the uncertainty of our estimate of the number of resistant cells at time *T*. Let us first calculate the variance of resistant cells at time *T* that originated from sensitive cells:

$$\begin{array}{c} \begin{array}{cc} & Var_{r}\left(X_{r}\left(T\right)\right)\\ \approx & \int_{0}^{T}\frac{r\left(t\right)\left(exp\left\{\int_{0}^{T-t}\left[\mu_{r}\left(\tau +t\right)-\lambda_{r}\left(\tau +t\right)\right]d\tau \right\}+2\int_{0}^{T-t}exp\left\{\int_{0}^{\tau }\left[\mu_{r}\left(\eta +t\right)-\lambda_{r}\left(\eta +t\right)d\eta \right]\lambda_{r}\left(\tau +t\right)\right\}d\tau \right)}{exp{\left\{\int_{0}^{T-t}\left[\mu_{r}\left(\tau +t\right)-\lambda_{r}\left(\tau +t\right)\right]d\tau \right\}}^{2}}dt \end{array} \end{array}$$ (7)

The variance of resistant cells originating from pre-existing resistant clones at time *T* is then given by

$$\begin{array}{c} \begin{array}{c} Var_{s}\left(X_{r}\left(T\right)\right)=X_{r}\left(0\right)\cdot exp\left\{-2exp\left\{\int_{0}^{\tau }\left[\mu_{r}\left(\eta \right)-\lambda_{r}\left(\eta \right)\right]d\eta \right\}\right\}\cdot \int_{0}^{t}\left[\lambda_{r}\left(\tau \right)+\mu_{r}\left(\tau \right)\right]exp\left\{\int_{0}^{\tau }\left[\mu_{r}\left(\eta \right)-\lambda_{r}\left(\eta \right)\right]d\eta \right\}d\tau . \end{array} \end{array}$$ (8)

Hence the total variance is given by *Var*(*X*_*r*_(*T*)) = *Var*_*s*_(*X*_*r*_(*T*)) + *Var*_*r*_(*X*_*r*_(*T*)).

### Estimating pharmacokinetic parameters and growth kinetics

Let us now estimate the pharmacokinetic parameters of erlotinib. We obtained data from pharmacokinetic studies from OSI/Astellas. In total, 28 subjects were given 100, 200, 400, 800, 1000, 1200, 1400, and 1600 mg of erlotinib, and the concentration of drug in the serum of these patients was measured before administration and at 2 hours, 8 hours and 24 hours after administration; see S1 Table for the data. We then employed an exponential decay function to model the concentration over time; this function is given by *C*(*t*) = *C*_*max*_ *e* ^−*κt*^, where *C*_*max*_ is the maximum concentration and *κ* is the elimination rate of the drug. Although both parameters vary across subjects, we used only one set of parameters for each dose for our primary results because of the relatively large sample size versus the small number of measured time points. The relationship between the dose amount and *C*_*max*_ was estimated, using least square methods, as *C*_*max*_(*d*) = 1.393 + 0.0198*d*. The rate *κ* = 0.05 per hour was estimated as the mean decay rate for the different dosing groups. Quantitative estimates of birth and death rates of sensitive and resistant cells were experimentally determined as in [33], using a pair of isogenic PC-9 human EGFR-mutant cell lines with and without the T790M point mutation that were treated with different concentrations of erlotinib. Using the cell counts of viable and dead cells, we then determined the exponential growth and death rates of the two cell types during different concentrations of the drug for use in the stochastic model outlined above and in [33], where *μ*_*X*_*s*__(*t*) ≈ 0.005 *hour* ^−1^, *μ*_*X*_*r*__(*t*) ≈ 0.002 *hour* ^−1^, *λ*_*X*_*s*__(*t*) ≈ *exp*(−4.4⋅*C*(*t*)−3.17) *hour* ^−1^, and *λ*_*X*_*r*__(*t*) ≈ −0.001⋅*C*(*t*) + 0.03 *hour* ^−1^.

All numerical calculations and simulations were coded in C++ and all statistical analyses were performed using open source R [43] software.

## Results

We then sought to validate our analytical approximations using exact stochastic computer simulations. As an example, let us consider the process specified in Eq (1) and define the birth and death rates for sensitive and resistant cells as follows:

$$\begin{array}{c} \begin{array}{cc} & \lambda_{X_{s}}\left(t\right)=A_{s}sin\left(\theta t\right)+B_{s},\;\;\;\;\;\;\;\;\mu_{X_{s}}\left(t\right)=C_{s},\\ & \lambda_{X_{r}}\left(t\right)=A_{r}sin\left(\theta t\right)+B_{r},\;\;\;\;\;\;\;\;\mu_{X_{r}}\left(t\right)=C_{r}. \end{array} \end{array}$$

These equations provide an example of a dose administration strategy leading to a drug concentration that varies like a sine function over time. Using this hypothetical treatment strategy, we then explored three different dependencies of the mutation rate on the dose: (1) a time-invariant mutation rate (independent of the drug concentration); (2) a mutation rate that increases with the drug concentration (or equivalently, decreasing with the birth rate); and (3) a mutation rate that decreases with the drug concentration (or equivalently, increasing with the birth rate). We can also interpret scenario (2) as a situation in which the sensitive cells do not have any delay in responding to the treatment effects of increasing the mutation rate, while scenario (3) represents a situation in which there is a one-period delay in the response. Thus we have

$$\begin{array}{c} \begin{array}{cc} & u_{independent}\left(t\right)=A_{u},\;\;\;\;\;\;\;\;u_{increasing}\left(t\right)=A_{u}cos\left(\theta t\right)+B_{u},\;\;\;\;\;\;\;\;u_{decreasing}\left(t\right)=A_{u}sin\left(\theta t\right)+B_{u}. \end{array} \end{array}$$

**Fig 2:**
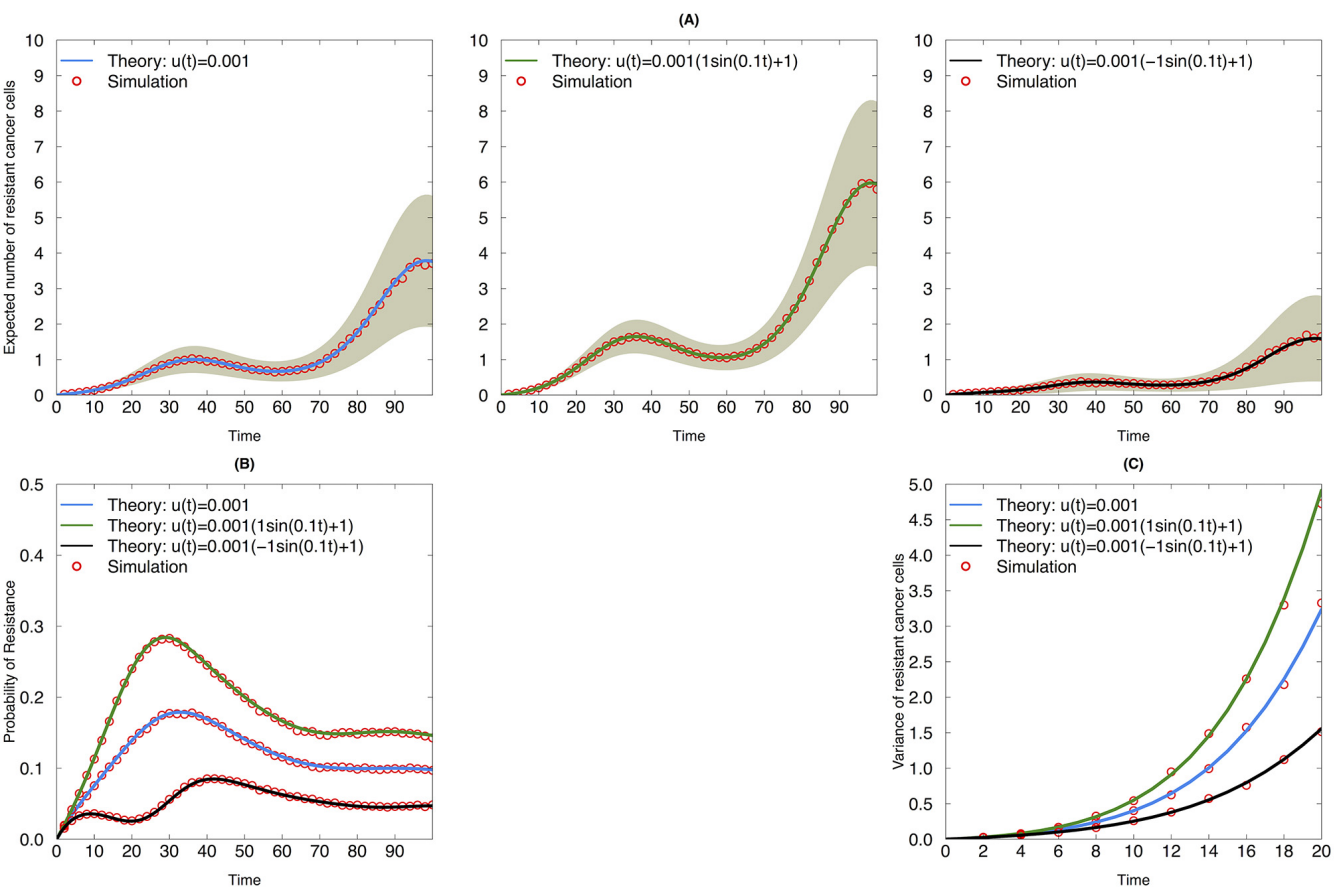
Evolutionary dynamics of sensitive and resistant clones during continuous therapy with different mutation dynamics. (A) Expected number of resistant cancer cells as a function of time during continuous therapy. Blue line: mutation rate is constant during treatment; black line: mutation rate increases with drug dose; green line: mutation rate decreases with drug dose. Red circles: simulation results. Grey shaded area indicates one analytic standard deviation from the analytic mean. (B) Probability of resistance as a function of time during continuous therapy. (C) Variance of resistant cancer cells as a function of time during continuous therapy. The following parametrizations were used for both simulation and analytic approximations: *A*_*s*_ = 0.05, *B*_*s*_ = 0.1, *C*_*s*_ = 0.005, *A*_*r*_ = 0.05, *B*_*r*_ = 0.12, *C*_*r*_ = 0.002, and *θ* = 0.10. The values for *A*_*u*_ and *B*_*u*_ are denoted in the panels for each corresponding scenario.

To validate the predictions of our analytical approximations, we first compared them with the output of exact stochastic computer simulations and observed good agreement (Fig 2). Our results show that the effects of the drug dose on the mutation rate (independent, increasing or decreasing mutation rate with drug concentration) influences the expected number of resistant cells (Fig 2A), the probability of resistance (Fig 2B), and the variance of resistant cells (Fig 2C). In particular, in this example, when the mutation rate changes in the opposite direction as the birth rate, as in the right panel of Fig 2A, the mutation rate increases with the drug concentration. When the mutation rate increases with the dose, the initially homogeneous tumor cell population is more prone to become heterogeneous as compared to the other scenarios. Nonetheless, the decreasing birth rate of the resistant cells impairs their growth. This fact is reflected in the lower expected number of resistant cells and probability of resistance over time.

**Fig 3:**
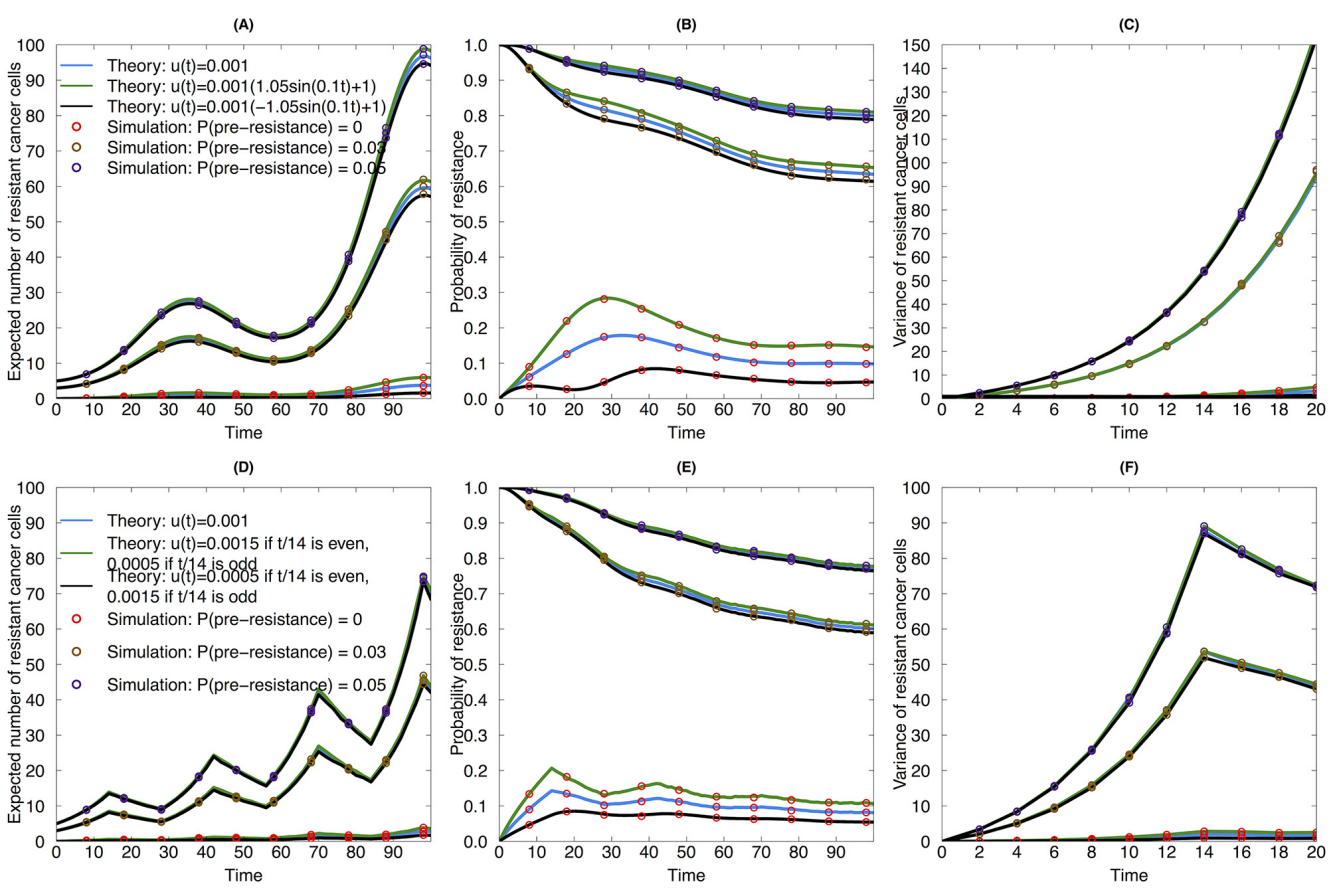
Evolutionary dynamics of sensitive and resistant clones during continuous therapy with pre-existing resistant cells. (A), (B) and (C) are examples for sine wave functional forms of birth, death and mutation rates. (A) Expected number of resistant cancer cells as a function of time during continuous therapy. Blue line: mutation rate is constant during treatment; black line: mutation rate increases with drug dose; green line: mutation rate decreases with drug dose. Red circles: simulation results for no pre-existing resistant clones; orange circle: simulation result for 3% proportion of pre-existing resistant clones; purple circle: simulation result for 5% proportion of pre-existing clones. (B) Probability of resistance as a function of time during continuous therapy. (C) Variance of resistant cancer cells as a function of time during continuous therapy. (D), (E), and (F) are examples for piecewise functional forms of birth, death and mutation rates. (D) Expected number of resistant cancer cells as a function of time during continuous therapy. (E) Probability of resistance as a function of time during continuous therapy. (F) Variance of resistant cancer cells as a function of time during continuous therapy. The death rates in Fig 2 were used here as the death rates for both upper and lower panels. The birth rates in the continuous therapy in Fig 2 were also used in this figure. The birth rates in the piecewise strategy were *λ*_*X*_*s*__(*t*) = 0.15⋅*I*(*t* / 14 mod 2 = 0) + 0.05⋅*I*(*t* / 14 mod 2 ≠ 0), *λ*_*X*_*r*__(*t*) = 0.17⋅*I*(*t* / 14 mod 2 = 0) + 0.07⋅*I*(*t* / 14 mod 2 ≠ 0).

We then considered a tumor cell population containing a proportion *s* of pre-existing resistant cells. We investigated a pulsed therapy schedule, where drug is administered for 14 days followed by a 14-day treatment holiday (step function), as well as the sine functional form of the dosing strategy. The step functions are given by:

$$\begin{array}{c} \begin{array}{cc} \lambda_{X_{s}}\left(t\right) & =0.15\cdot I(t/14\;\text{mod}\;2=0)+0.05\cdot I(t/14\;\text{mod}\;2\neq 0),\\ \lambda_{X_{r}}\left(t\right) & =0.17\cdot I(t/14\;\text{mod}\;2=0)+0.07\cdot I(t/14\;\text{mod}\;2\neq 0),\\ u_{independent}\left(t\right) & =0.001,\\ u_{increasing}\left(t\right) & =0.0005\cdot I(t/14\;\text{mod}\;2=0)+0.0015\cdot I(t/14\;\text{mod}\;2\neq 0),\\ u_{decreasing}\left(t\right) & =0.0015\cdot I(t/14\;\text{mod}\;2=0)+0.0005\cdot I(t/14\;\text{mod}\;2\neq 0), \end{array} \end{array}$$

where *I*(⋅) denotes the indicator function. In this case, the death rate is held as a constant as in Fig 2, while the mutation rate is assumed to be either constant, changing in the same direction, or in the opposite direction as the birth rate. We again compared our theoretical approximations to the results of the exact stochastic simulations and found good agreement (Fig 3). The same observation as in Fig 2 can be obtained when (1) there is a pre-existing resistant clone and (2) a step function of birth, death and mutation rates is chosen: when the mutation rate changes in the opposite direction as the dosing regime, then the tumor cells are more likely to develop a resistance mutation as compared to the other two scenarios, i.e. the time-invariant mutation rate and the mutation rate increasing with the drug concentration. Interestingly, we noticed that when there are pre-existing resistant clones, the impact of the dose-dependent mutation rate is not as remarkable as when the initial cell population is homogeneous.

Next, we investigated the pharmacokinetic effects of drug accumulation *in vivo*. From this point on, all results were obtained from analytic approximation, which has been proved to be consistent with the exact simulation in the previous sections. We modeled the drug elimination in the human system after one dose as an exponential decay with rate *κ*. When the dosing amount is given as *D* with corresponding *C*_*max*_ using the equation developed in the **Methods** section, then the drug concentration in the body is given by *Concentration*(*t*) = *C*_*max*_ *e* ^−*κt*/*T*^, where *T* is the dosing interval. Note that the drug concentration reaches a steady state around *C*_*max*_/[1−*e* ^−*κt*/*T*^] after a sufficiently long time.

**Fig 4:**
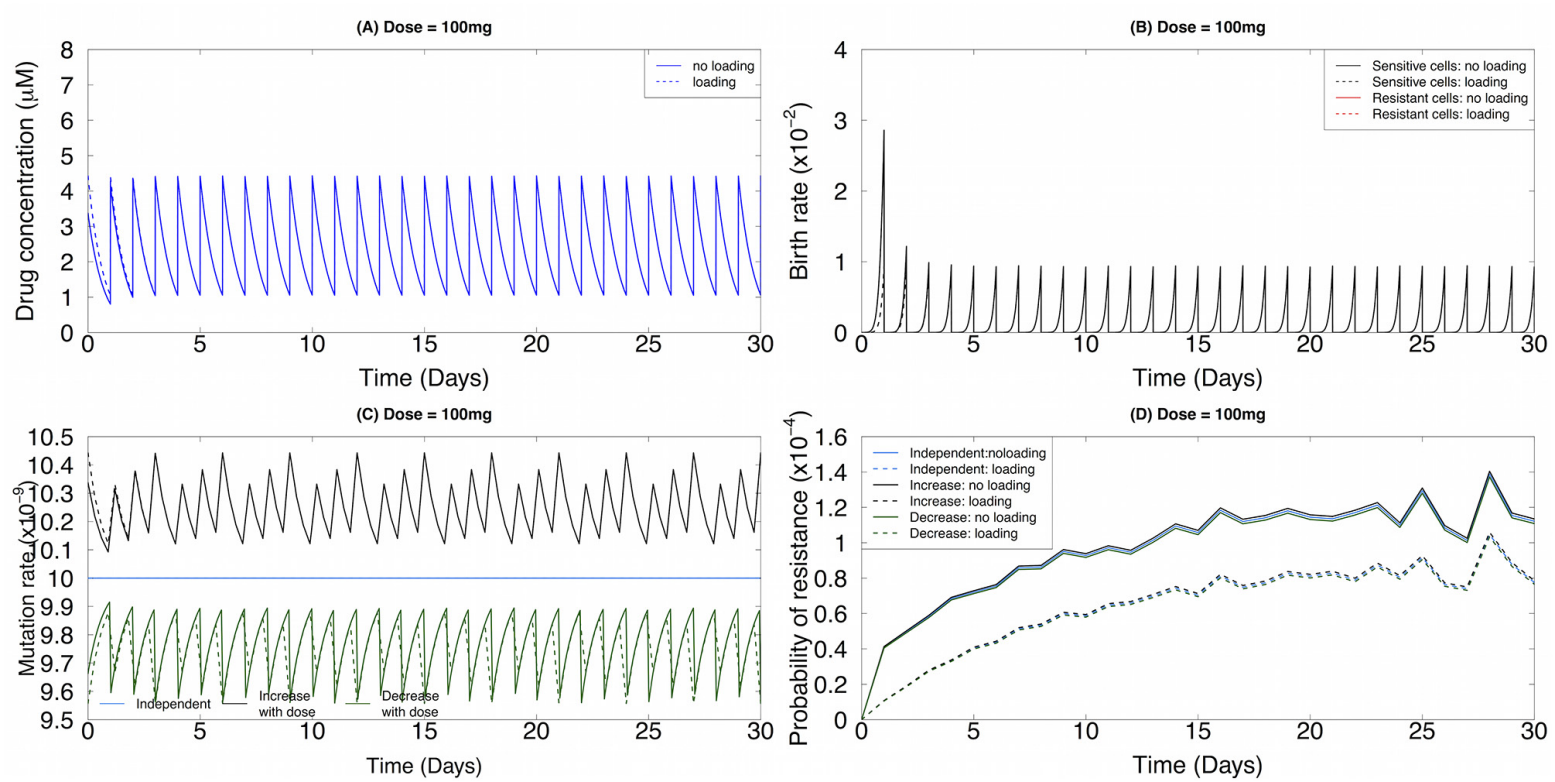
Pharmacokinetic effects influence the evolutionary dynamics of resistance. Here we consider a baseline mutation rate per cell division of 10^−8^ and the effect of drug concentration on the mutation rate *β* = 10^−9^. (A) The drug concentration *in vivo* based on the pharmacokinetic model over time for a 100mg per day continuous dosing regime. Dotted line: loading dose; solid line: no loading dose. (B) The birth rates as a function of time *t*. Red line: the birth rate of the resistant cells; black line: the birth rate of the sensitive cells. (C) The mutation rate of sensitive cells as a function of time *t*. Blue line: constant mutation rate; green line: mutation rate monotonically decreases with the drug concentration; black line: mutation rate monotonically increases with the drug concentration. (D) The probability of resistance as a function of time *t*. Values for birth and death rates: *μ*_*X*_*s*__(*t*) ≈ 0.005 *hour* ^−1^, *μ*_*X*_*r*__(*t*) ≈ 0.002 *hour* ^−1^, *λ*_*X*_*s*__(*t*) ≈ *exp*(−4.4 ⋅ *C*(*t*)−3.17) *hour* ^−1^, and *λ*_*X*_*r*__(*t*) ≈ −0.001 ⋅ *C*(*t*) + 0.03 *hour* ^−1^.

A loading dose is sometimes used to cause the drug concentration to reach its steady level more quickly. We thus compared the effects of different mutation rate scenarios (independent, increasing and decreasing rates with dose) for drug administration schedules with and without loading doses. The birth and death rates of non-small cell lung cancer cells as a function of the erlotinib concentration were estimated as in [33]; the birth rate is a linear function of the drug concentration and the death rate is given by an exponential decay of the drug concentration. All coefficients were obtained through least square estimation. The dosing regime in terms of drug concentration *in vivo* and the related birth and death rates are displayed in Fig 4A and 4B, respectively. We considered a scenario in which there are 10^6^ tumor (“stem”) cells capable of propagating the entire tumor cell population, with no pre-existing resistant cells. For each dosing strategy, we again considered three different functional forms for the mutation rate: (a) the mutation rate is independent of the drug concentration and hence is fixed throughout treatment, *u*(*t*) = *u*_0_; (b) the mutation rate linearly increases with the drug concentration, *u*(*t*) = *β* ⋅ *Concentration*(*t*) + *u*_0_; and (c) the mutation rate linearly decreases with the drug concentration: *u*(*t*) = −*β* ⋅ *Concentration*(*t*) + *u*_0_, where *β* > 0 denotes the effect of the drug concentration on the mutation rate *in vivo*, while *u*_0_ is the baseline mutation rate, chosen as *u*_0_ = 10^−8^ (Fig 4C). Here we controlled the independent mutation rate as the time-average of the mutation rate increasing (or decreasing) with the drug concentration. The probability of resistance over time for these scenarios is shown in Fig 4D. In all cases, a dosing strategy with a loading dose outperforms the strategy without a loading dose. Based on the current parametrization, the lowest probability of resistance was obtained in the case in which the mutation rate monotonically increases with the drug concentration, suggesting a different treatment performance under different mutation rate assumptions. To test the robustness of our results, we performed similar analyses with *β* = 10^−9^ (S1 Fig) and found robust results. Together, these findings demonstrate that the magnitude of the mutation rate does not change the relative order of the performance of the three mutation regimes (a)–(c): the mutation rate linearly increasing with dose delivers the lowest probability of emerging resistance.

**Fig 5:**
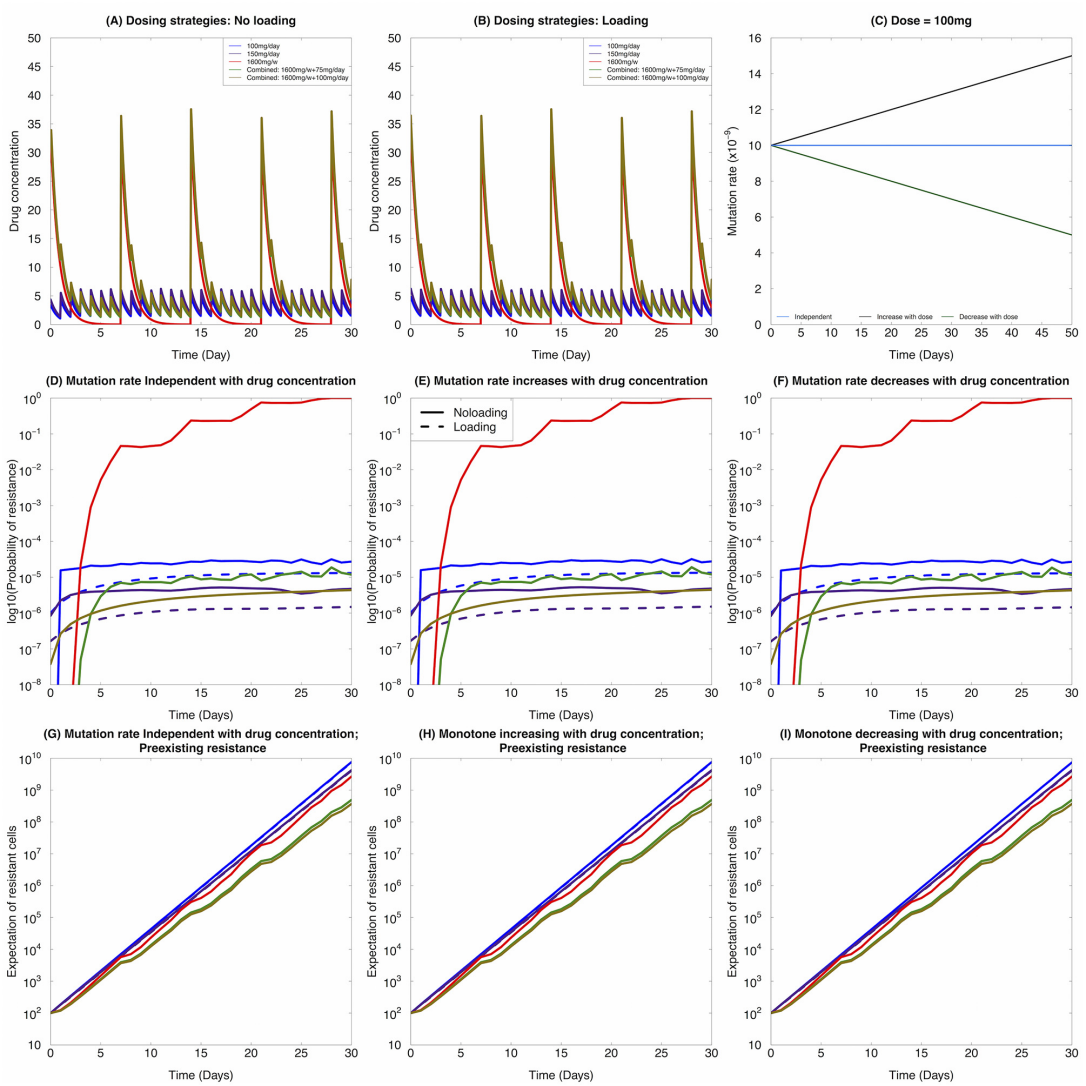
Effects of varying dosing regimes on the evolution of resistance. Here we consider *β* = 10^−10^. (A) The dosing regimes with no loading dose for 100mg/day, 150mg/day, 1600mg/week, 1600mg/week combined with 100mg/day during the week, and 1600mg/week combined with 75mg/day during the week. (B) The dosing regimes with loading dose for 100mg/day, 150mg/day, 1600mg/week, 1600mg/week combined with 100mg/day during the week, and 1600mg/week combined with 75mg/day during the week. (C) Mutation rate as a function of treatment concentration under different assumptions: blue: independent with treatment concentration; black: increasing with treatment concentration; green: decreasing with treatment concentration. (D)-(F) Without pre-existing resistance, the probability of resistance monitored up to one month under (D) constant mutation rate, (E) mutation rate increasing with the drug concentration, and (F) mutation rate decreasing with the drug concentration. (G)-(I) With pre-existing resistance, the expected number of resistant cells monitored up to one month under (G) constant mutation rate, (H) mutation rate increasing with the drug concentration, and (I) mutation rate decreasing with the drug concentration. Dotted line: with loading dose; solid line: without loading dose. Values for birth and death rates: *μ*_*X*_*s*__(*t*) ≈ 0.005 *hour* ^−1^, *μ*_*X*_*r*__(*t*) ≈ 0.002 *hour* ^−1^, *λ*_*X*_*s*__(*t*) ≈ *exp*(−4.4 ⋅ *C*(*t*)−3.17) *hour* ^−1^, and *λ*_*X*_*r*__(*t*) ≈ −0.001 ⋅ *C*(*t*) + 0.03 *hour* ^−1^

We then compared the performance of different dosing regimes under the three different types of dynamic mutation rate assumptions as described above. These dosing regimes include: 100mg/day, 1600mg/week, and 1600mg/week combined with 100mg/day, together with loading and no loading doses. The dosing regimes as a function of time *t* are shown in Fig 5A and 5B. The mutation rates over time under different assumptions are displayed in Fig 5C. Overall, when comparing across different mutation rate assumptions, without loading doses, we found that 150mg/day and the combined strategy of 1600mg/week + 100mg/day lead to the lowest probability of resistance without pre-existing resistant clones (Fig 5D–5F). When a loading dose is given, the relative performance of different strategies is altered, especially between the 150mg/day and combined strategies, where 150mg/day outperformed the combined strategy under all mutation rates assumptions. When there are pre-existing resistant clones, however, the presence of a loading dose did not significantly alter the dynamics (Fig 5G–5I). Finally, the different mutation rates assumptions also did not have an effect of the relative performance of different dosing schedules. Fig 5 displays results where *β* = 10^−10^, and similar results are shown in S2 Fig for *β* = 10^−9^ to corroborate our conclusion: within the scenarios tested, different mutation rates do not alter the relative performance of different dosing schedules.

Next, we took pre-existing resistance into consideration, assuming that there are 100 pre-existing resistant cells in the tumor. We then used the expected number of resistant cells in the population as a proxy for evaluating the performance of each dosing strategy. In this situation, different mutation rate assumptions did not lead to significantly different performance results of different dosing regimes (Fig 5F–5H): the combined therapy leads to the best performance under all three different assumptions.

**Fig 6:**
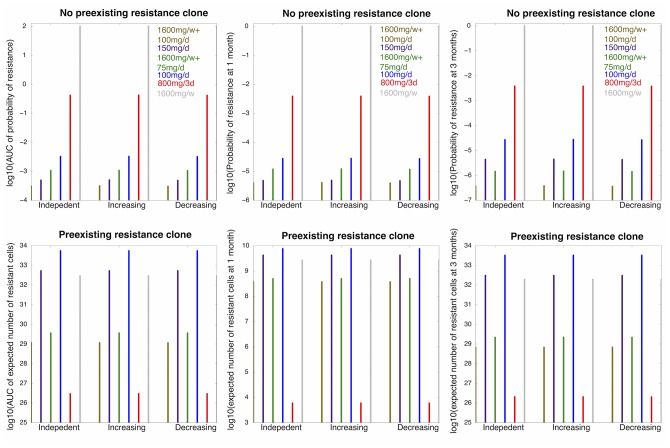
The optimum dosing strategy varies with dose-dependent mutation rates. Here we consider *β* = 10^−10^. (A)–(C): (A) The area under the curve (AUC) of the probability of resistance over three months of treatment, (B) the probability of resistance after one month of treatment, and (C) the probability of resistance after three months of treatment when there are no pre-existing resistant cells for different treatment schedules (separated by different colors) and different mutation rate assumptions (independent; increasing with drug concentration; decreasing with drug concentration) indicated by the x-axis label. (D)–(F): (D) The AUC of the expected number of resistant cells over three months of treatment, (E) the expected number of resistant cells after one month of treatment, and (F) the expected number of resistant cells after three months of treatment when there are pre-existing resistant clones for different treatment schedules (separated by different colors) and different mutation rate assumptions (independent; increasing with drug concentration; decreasing with drug concentration) indicated by the x-axis label. Values for birth and death rates: *μ*_*X*_*s*__(*t*) ≈ 0.005 *hour* ^−1^, *μ*_*X*_*r*__(*t*) ≈ 0.002 *hour* ^−1^, *λ*_*X*_*s*__(*t*) ≈ *exp*(−4.4 ⋅ *C*(*t*)−3.17) *hour* ^−1^, and *λ*_*X*_*r*__(*t*) ≈ −0.001 ⋅ *C*(*t*) + 0.03 *hour* ^−1^.

Finally, we considered the optimum dosing strategy for the three different forms of mutation rate functions discussed above under the constraint of side effects and toxicity. We used a similar constraint as derived in [32], where *ToleratedDose*(*Freq*) = 50*exp*(−0.4 × *Freq*). For each dosing frequency corresponding to the dosing amount (100mg, 800mg, 1600mg) explored in our study, we rounded them to the unit of half day. In addition, the FDA-approved 150mg/day dosing regime and two combined strategies 1600mg/week + 75mg/day and 1600mg/week + 100mg/day were also included in the analysis. Only schedules with no loading dose were explored. We then monitored the following measures as the utilities of each dosing scheme: (i) the area under the curve of the trajectory of probability of resistance, (ii) the area under the curve of the expected number of resistant cell over time, (iii) the probability of resistance and (iv) the expected number of resistant cells after one and three months of treatment (Fig 6 and S3 Fig). In all cases, different assumptions of the mutation rates did not change the ranking of the dosing schemes based on either of the different measures.

## Discussion

In this study, we have built a mathematical framework by using a non-homogeneous multi-type birth-and-death process model with dosing-dependent mutation rates to investigate the evolution of resistant clones to targeted cancer therapies. The consideration of dose-dependent mutation rates can lead to different stochastic dynamics of the sensitive and resistant cancer cell populations as compared to the dose-independent case [44]. Thus, its inclusion might lead to different dosing strategies that are best able to prevent or delay the emergence of the resistant cell population. Throughout this paper, we have also assumed that the drug concentration inside the tumor is uniform since there is insufficient data available to parameterize other functional forms. Should the drug concentration be more uneven across the tumor, then regions with lower concentration might lead to different rates of generating resistant cells. Such conditions will be analyzed when more data becomes available. We applied our mathematical framework to non-small cell lung cancer treated in particular with the the targeted agent erlotinib to identify best dosing strategies for this cancer type; we found that in this application, different considerations about the relationship between the mutation rate and the drug concentration do not change the finding that resistance is optimally delayed using a low dose continuous plus high dose pulsed approach [33]. This strategy is currently being tested as a prospective clinical trial at Memorial Sloan-Kettering Cancer Center (NCT01967095). While this clinical trial of our original optimized dosing strategy (see Chmielecki et al, Science Translational Medicine 2011 [33]) is ongoing, the current work is meant to provide a more in-depth analysis of situations in which the drug concentration might influence the kinetics of mutation acquisition, in order to evaluate whether such scenarios might change the optimum dosing strategies. No direct clinical validation is currently possible.

Currently, the emergence of resistant cells is attributed to clonal expansion of pre-existing populations of cells that harbor such resistance mutations [45–50]. For example, EGFR T790M can be detected at low levels in patients with EGFR-mutant NSCLC prior to treatment [50]. However, an alternative explanation is that kinase inhibitor treatment indirectly induces resistance through DNA damage incurred as a result of increased cell killing and subsequent release of ROS [34, 35]. To account for this possible scenario, our mathematical framework incorporates non-homogeneous multi-type birth-and-death processes with a dosing-dependent mutation rate to investigate the evolution of resistant clones to targeted cancer therapies under general dosing regimens. We assume that a dose-dependent mutation rate may produce different stochastic dynamics of sensitive and resistant cancer cell population as compared to the dose-independent case, and thus may suggest different dosing strategies to prevent or at least slow down the emergence of the resistant cell population. In addition to the T790M mutation, cells might accumulate other genetic and/or epigenetic changes that might change the fitness of the cell. To address this situation, in our model, we implicitly assume that each cell population has a distribution of growth rates and death rates, and the kinetically dominant subpopulation of each mutation type (sensitive those cells that do not harbor the T790M resistance mutation but potentially other alterations, and resistant those cells that do harbor the T790M resistance mutation and also potentially other changes) drives the outgrowth of such clones. Specifically, this new model uses detailed pharmacokinetic data to examine three possible clinical scenarios involving EGFR mutant lung cancer treated with erlotinib: mutation rates are independent of treatment, increase with treatment, or decrease with treatment. In all situations considered, including pre-existing or no pre-existing resistant clones, and loading or no loading dose in the beginning of the treatment schedules, the relative performance of different dosing strategies is robust against assumptions of mutation rates. These findings suggest that considering constant mutation rate is sufficient for modeling the effects of different dosing strategies in EGFR-mutant lung cancer treated with erlotinib. Currently, little data is available for cell kinetics in response to DNA damaging agents and therefore we have only focused on the treatment response to erlotinib in this paper. However, our modeling framework can be applied to DNA damaging agents as well as long as the relevant growth and death rates are available.

For more precise parameterization of our model, time-series data of the growth, death and mutation rates during different drug combinations and doses are required. Next-generation sequencing has been widely used to determine the mutation frequencies under different treatments or cancer subtypes [51], but it will require a very large sequencing depth and information on the total number of cell divisions to obtain estimates for mutation rates per cell division. Future studies will attempt to confirm our models based upon actual mutation rate data induced by inhibitor treatment. Our model can also be extended to investigate combination drug therapy, which has been suggested as a possible solution for overcoming resistance to cancer therapy [52].

## Supporting Information

S1 Fig Pharmacokinetic effects influence the evolution of resistance. Here we consider the baseline mutation rate per cell division to be 10^−8^ and the effect of drug dose on the mutation rate to be *β* = 10^−10^. (A) The drug concentration *in vivo* based on the pharmacokinetic model over time for 100mg per day dosing regime. Dotted line: loading dose; solid line: no loading dose. (B) The birth rates as a function of time *t*. Red line: the birth rate of the resistant cells; black line: the birth rate of the sensitive cells. (C) The mutation rate of sensitive cells as a function of time *t*. Blue line: constant mutation rate; green line: mutation rate monotonically decreases with the drug concentration; black line: mutation rate monotonically increases with the drug concentration. (D) The probability of resistance as a function of time *t*. Values for birth and death rates: *μ*_*X*_*s*__(*t*) ≈ 0.005 *hour* ^−1^, *μ*_*X*_*r*__(*t*) ≈ 0.002 *hour* ^−1^, *λ*_*X*_*s*__(*t*) ≈ *exp*(−4.4 ⋅ *C*(*t*)−3.17) *hour* ^−1^, and *λ*_*X*_*r*__(*t*) ≈ −0.001 ⋅ *C*(*t*) + 0.03 *hour* ^−1^.
(TIFF)

S2 Fig Effects of varying dosing regimes on the evolution of resistance. Here we consider *β* = 10^−9^. (A) The dosing regimes with no loading dose for 100mg/day, 150mg/day, 1600mg/week, 1600mg/week combined with 100mg/day during the week, and 1600mg/week combined with 75mg/day during the week. (B) The dosing regimes with loading dose for 100mg/day, 150mg/day, 1600mg/week, 1600mg/week combined with 100mg/day during the week, and 1600mg/week combined with 75mg/day during the week. (C) Mutation rate as a function of treatment concentration under different assumptions: blue: independent with treatment concentration; black: increasing with treatment concentration; green: decreasing with treatment concentration. (D)-(F) Without pre-existing resistance, the probability of resistance monitored up to one month under (D) constant mutation rate, (E) mutation rate increasing with the drug concentration, and (F) mutation rate decreasing with the drug concentration. (G)-(I) With pre-existing resistance, the expected number of resistant cells monitored up to one month under (G) constant mutation rate, (H) mutation rate increasing with the drug concentration, and (I) mutation rate decreasing with the drug concentration. Dotted line: with loading dose; solid line: without loading dose. Values for birth and death rates: *μ*_*X*_*s*__(*t*) ≈ 0.005 *hour* ^−1^, *μ*_*X*_*r*__(*t*) ≈ 0.002 *hour* ^−1^, *λ*_*X*_*s*__(*t*) ≈ *exp*(−4.4 ⋅ *C*(*t*)−3.17) *hour* ^−1^, and *λ*_*X*_*r*__(*t*) ≈ −0.001 ⋅ *C*(*t*) + 0.03 *hour* ^−1^.
(TIFF)

S3 Fig The optimum dosing strategy varies with dose-dependent mutation rates. Here we assume *β* = 10^−9^. (A)–(C): (A) The AUC of probability of resistance over three months of treatment, (B) the probability of resistance after one month of treatment, and (C) the probability of resistance after three months of treatment when there are no preexisting resistant clones for different treatment schedules (separated by different colors) and different mutation rate assumptions (independent; increasing with drug concentration; decreasing with drug concentration) indicated by the x-axis label. (D)–(F): (D) The AUC of expected number of resistant cells over three months of treatment, (E) the expected number of resistant cells after one month of treatment, and (F) the expected number of resistant cells after three months of treatment when there are preexisting resistant clones for different treatment schedules (separated by different colors) and different mutation rate assumptions (independent; increasing with drug concentration; decreasing with drug concentration) indicated by the x-axis label. Values for birth and death rates: *μ*_*X*_*s*__(*t*) ≈ 0.005 *hour* ^−1^, *μ*_*X*_*r*__(*t*) ≈ 0.002 *hour* ^−1^, *λ*_*X*_*s*__(*t*) ≈ *exp*(−4.4 ⋅ *C*(*t*)−3.17) *hour* ^−1^, and *λ*_*X*_*r*__(*t*) ≈ −0.001 ⋅ *C*(*t*) + 0.03 *hour* ^−1^.
(TIFF)

S1 Table Pharmacokinetic data for the concentration of erlotinib in the serum of 28 patients from OSI/Astellas study. *T*_*max*_ denotes the average maximum of time to reach *C*_*max*_, where *C*_*max*_ is the maximum concentration measured in the serum. The patients are followed at 2 hours, 8 hours, and 24 hours after erlotinib delivery.
(XLSX)
