# Supplementary figures and images for: Dose-Dependent Mutation Rates Determine Optimum Erlotinib Dosing Strategies for EGFR Mutant Non-Small Cell Lung Cancer Patients
Source: PLoS One. 2015 Nov 4;10(11):e0141665. doi: 10.1371/journal.pone.0141665 (PMC4633116; doi:10.1371/journal.pone.0141665)

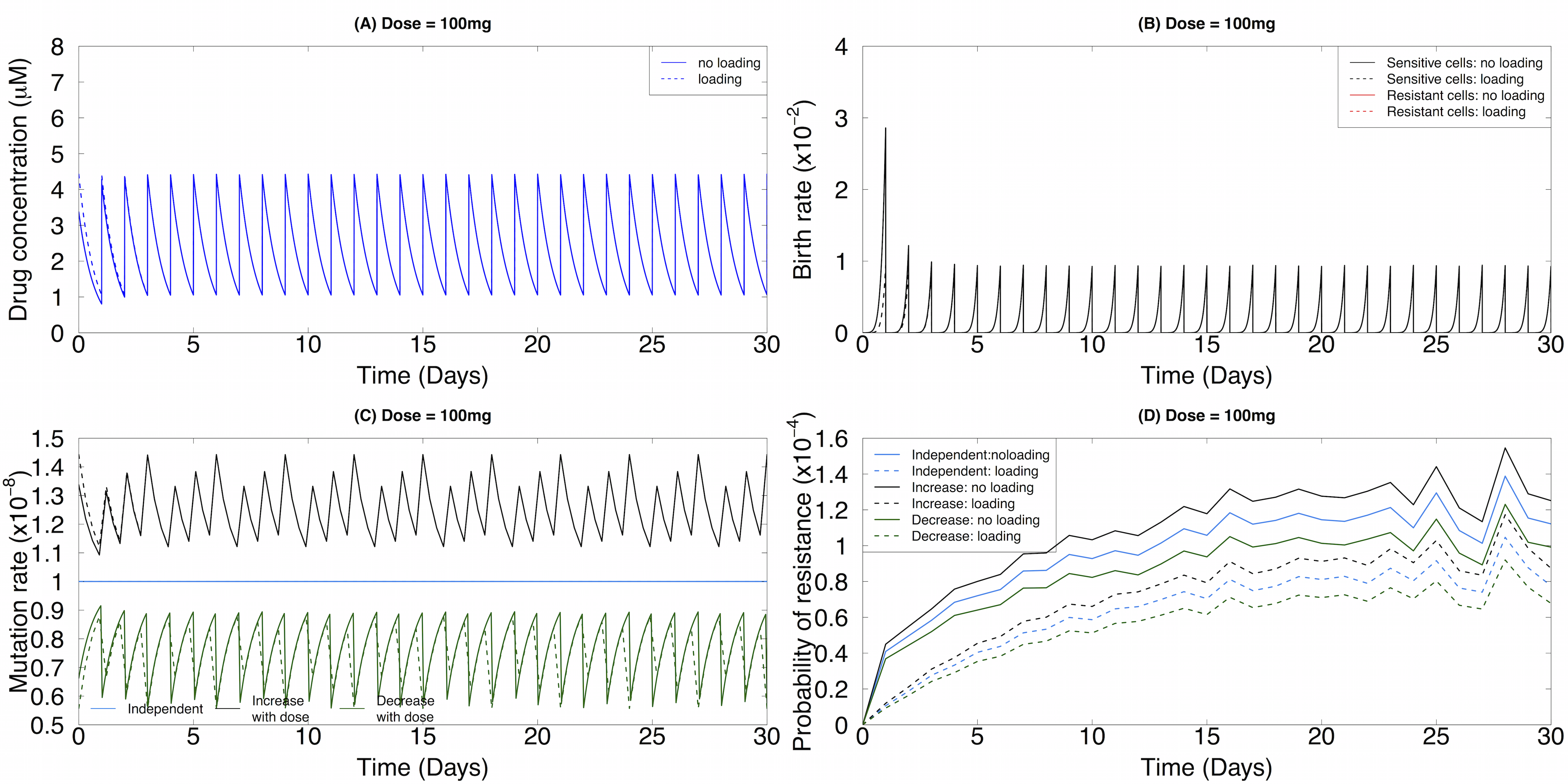

Supplement: S1 Fig — Here we consider the baseline mutation rate per cell division to be 10−8 and the effect of drug dose on the mutation rate to be β = 10−10. (A) The drug concentration in vivo based on the pharmacokinetic model over time for 100mg per day dosing regime. Dotted line: loading dose; solid line: no loading dose. (B) The birth rates as a function of time t. Red line: the birth rate of the resistant cells; black line: the birth rate of the sensitive cells. (C) The mutation rate of sensitive cells as a function of time t. Blue line: constant mutation rate; green line: mutation rate monotonically decreases with the drug concentration; black line: mutation rate monotonically increases with the drug concentration. (D) The probability of resistance as a function of time t. Values for birth and death rates: μ Xs(t) ≈ 0.005 hour −1, μ Xr(t) ≈ 0.002 hour −1, λ Xs(t) ≈ exp(−4.4 ⋅ C(t)−3.17) hour −1, and λ Xr(t) ≈ −0.001 ⋅ C(t) + 0.03 hour −1. (TIFF) [file pone.0141665.s001.tiff]

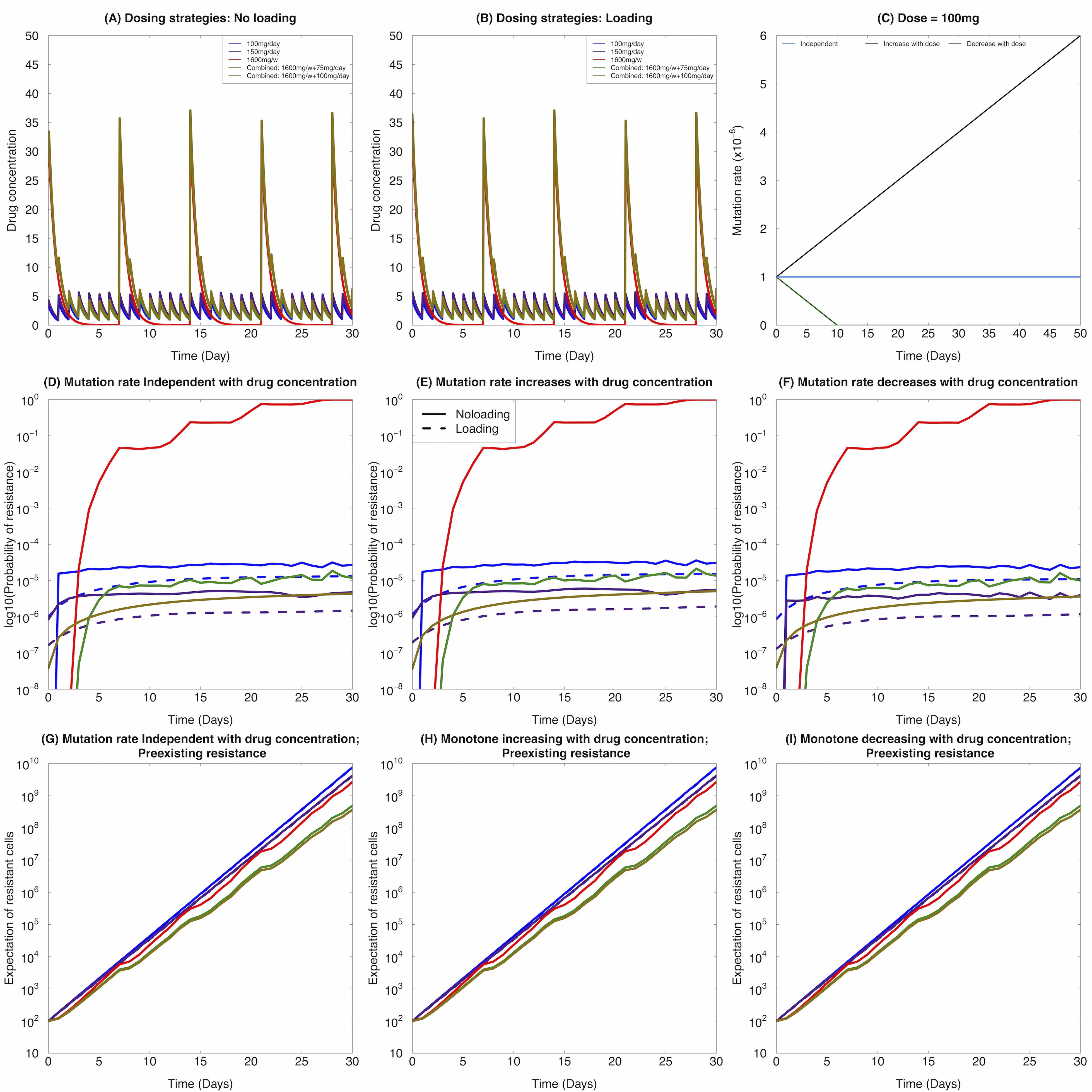

Supplement: S2 Fig — Here we consider β = 10−9. (A) The dosing regimes with no loading dose for 100mg/day, 150mg/day, 1600mg/week, 1600mg/week combined with 100mg/day during the week, and 1600mg/week combined with 75mg/day during the week. (B) The dosing regimes with loading dose for 100mg/day, 150mg/day, 1600mg/week, 1600mg/week combined with 100mg/day during the week, and 1600mg/week combined with 75mg/day during the week. (C) Mutation rate as a function of treatment concentration under different assumptions: blue: independent with treatment concentration; black: increasing with treatment concentration; green: decreasing with treatment concentration. (D)-(F) Without pre-existing resistance, the probability of resistance monitored up to one month under (D) constant mutation rate, (E) mutation rate increasing with the drug concentration, and (F) mutation rate decreasing with the drug concentration. (G)-(I) With pre-existing resistance, the expected number of resistant cells monitored up to one month under (G) constant mutation rate, (H) mutation rate increasing with the drug concentration, and (I) mutation rate decreasing with the drug concentration. Dotted line: with loading dose; solid line: without loading dose. Values for birth and death rates: μ Xs(t) ≈ 0.005 hour −1, μ Xr(t) ≈ 0.002 hour −1, λ Xs(t) ≈ exp(−4.4 ⋅ C(t)−3.17) hour −1, and λ Xr(t) ≈ −0.001 ⋅ C(t) + 0.03 hour −1. (TIFF) [file pone.0141665.s002.tiff]

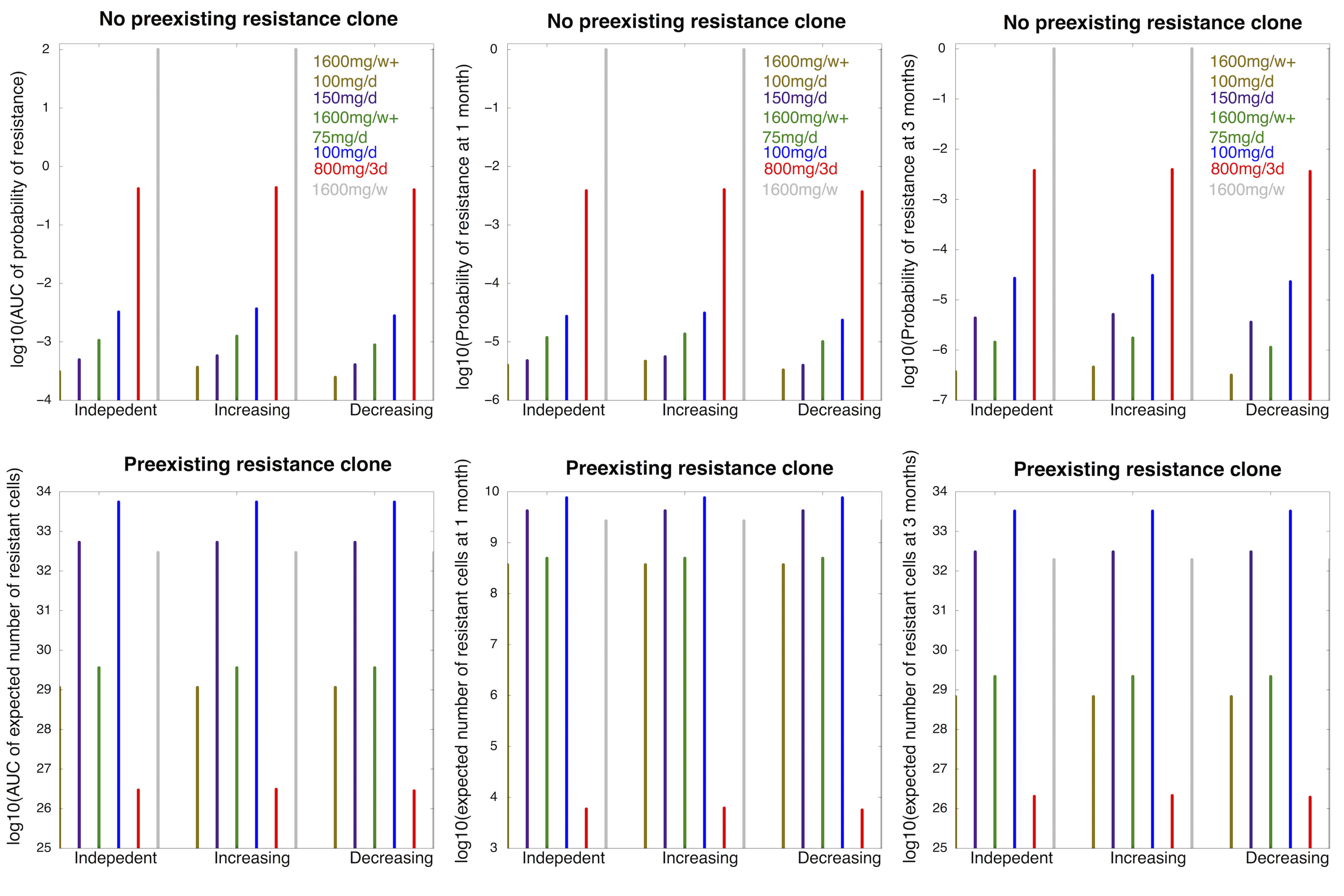

Supplement: S3 Fig — Here we assume β = 10−9. (A)–(C): (A) The AUC of probability of resistance over three months of treatment, (B) the probability of resistance after one month of treatment, and (C) the probability of resistance after three months of treatment when there are no preexisting resistant clones for different treatment schedules (separated by different colors) and different mutation rate assumptions (independent; increasing with drug concentration; decreasing with drug concentration) indicated by the x-axis label. (D)–(F): (D) The AUC of expected number of resistant cells over three months of treatment, (E) the expected number of resistant cells after one month of treatment, and (F) the expected number of resistant cells after three months of treatment when there are preexisting resistant clones for different treatment schedules (separated by different colors) and different mutation rate assumptions (independent; increasing with drug concentration; decreasing with drug concentration) indicated by the x-axis label. Values for birth and death rates: μ Xs(t) ≈ 0.005 hour −1, μ Xr(t) ≈ 0.002 hour −1, λ Xs(t) ≈ exp(−4.4 ⋅ C(t)−3.17) hour −1, and λ Xr(t) ≈ −0.001 ⋅ C(t) + 0.03 hour −1. (TIFF) [file pone.0141665.s003.tiff]
